# Evaluating large-scale propensity score adjustment when sample size is small

**DOI:** 10.1093/jamia/ocag133

**Published:** 2026-08-04

**Authors:** Fleur Vereijken, Jenna M Reps, Marc A Suchard, Linying Zhang, George Hripcsak, Peter R Rijnbeek, Ross D Williams, Martijn J Schuemie

**Affiliations:** Department of Medical Informatics, Erasmus University Medical Center, Rotterdam, 3015 GD, The Netherlands; Department of Medical Informatics, Erasmus University Medical Center, Rotterdam, 3015 GD, The Netherlands; Observational Health Data Analytics, Janssen Research and Development, Titusville, NJ, 08560, United States; Department of Biostatistics, University of California, Los Angeles, Los Angeles, CA 90095-1772, United States; Data Science and Biostatistics, Washington University School of Medicine, Saint Louis, Missouri, MO 63110, United States; Department of Biomedical Informatics, Columbia University Medical Center, New York, NY 10032, United States; Department of Medical Informatics, Erasmus University Medical Center, Rotterdam, 3015 GD, The Netherlands; Department of Medical Informatics, Erasmus University Medical Center, Rotterdam, 3015 GD, The Netherlands; Observational Health Data Analytics, Janssen Research and Development, Titusville, NJ, 08560, United States; Department of Biostatistics, University of California, Los Angeles, Los Angeles, CA 90095-1772, United States

**Keywords:** Confounding adjustment, Propensity score, distributed data network, small sample size, real-world data

## Abstract

**Background:**

Large-scale propensity score (LSPS) models are increasingly used to control confounding in observational studies, but their reliability in small-sample settings is unclear. Small samples can limit a study’s ability to estimate propensity scores accurately and achieve adequate covariate balance, raising concerns about insufficient confounding adjustment. Resulting in small datasets being excluded, despite potentially containing valid information.

**Methods:**

We evaluated the balancing and bias reduction performance of LSPS models using six target-comparator pairs. To emulate a distributed data network analysis setting, we partitioned two large real-world data sources into smaller subsets. Within each subset, LSPS models were fit locally and evaluated on covariate balance, treatment effect bias and precision after empirical calibration, using real negative controls and synthetic positive controls. Performance was compared to a global LSPS model trained on the full dataset.

**Results:**

Across most target-comparator pairs, LSPS models substantially reduced bias and, when applying empirical calibration, improved precision. PS matching was more resilient to small sample sizes than PS stratification, achieving adequate balance even at *n* = 500, while stratification sometimes failed at *n* = 4000. A slight bias increase was observed at the smallest sample sizes, though not universally. Standard balance diagnostics consistently failed below *n* = 20 000, while a recently proposed diagnostic accounting for chance imbalance did not.

**Conclusions:**

LSPS models generally provide reliable bias reduction in small-sample settings, supporting their use in federated analyses. However, standard balance diagnostics may be misleading in small samples, and alternatives should be considered, such as significance checking. When LSPS fails to reduce bias adequately, additional adjustment strategies are required.

## Introduction

Estimating treatment effects from observational data is a foundational task in comparative effectiveness research. While randomized controlled trials (RCTs) are often considered the gold standard due to the benefits of randomization in balancing treatment groups, they are not always feasible due to practical constraints such as ethical, financial, or logistical considerations, and are often underpowered for safety analysis.^1^^,^^2^ Observational studies, for example, using existing healthcare data such as electronic health records and administrative claims, offer an alternative to complement RCTs. However, in observational studies, treatment is not assigned at random, creating the risk of confounding, where differences in outcomes are partly explained by differences in baseline characteristics. Statistical methods can control for measured confounders. Yet, when important covariates are unknown, unmeasured, or poorly measured, residual confounding may remain and bias observed treatment effect.^3^^,^^4^ Addressing confounding is essential to draw valid causal inferences.

A common approach to address confounding in observational studies is the use of propensity scores (PS). A PS estimates the probability of receiving a particular treatment based on observed baseline characteristics. These scores can then be used in methods such as matching or inverse probability weighting. The goal is to create treatment groups that are comparable in their measured characteristics, similar to what randomization achieves in RCTs. By improving covariate balance between treatment groups, PS methods help isolate the treatment effect.^5^^,^^6^ In some contexts, these methods may yield more generalizable or granular insights than small underpowered RCTs.^7^^,^^8^

Traditional PS methods have been widely studied and validated in large-sample settings. However, their performance in small datasets has raised methodological concerns. Small sample sizes can lead to unstable effect estimates, poor covariate balance, and increased bias.^9^ Several studies show that, when models include true confounders and outcome predictors, PS methods can still give reliable results in smaller cohorts. For instance, Pirracchio et al.^10^ demonstrated using both empirical and simulated data that valid estimates are achievable in small datasets when PS models rely on a limited set of clinically informed covariates that are strong confounders. Similarly, Bottigliengo et al.^9^ showed that selecting key variables, using traditional PS methods, along with strategies such as oversampling and matching with replacement, can improve covariate balance and effect estimation performance in small samples.

Recently, large-scale propensity score (LSPS) models have emerged as an extension of traditional PS methods, particularly suited to high-dimensional observational health data networks.^11^ Traditional PS models typically include a small number of covariates selected based on subject-matter expertise, tailored to the specific study question to adjust for confounding. In contrast, LSPS models automate the inclusion of a vast number of covariates, often tens of thousands. These covariates are generic, meaning the same comprehensive set is used across studies, regardless of the specific research question. LSPS models rely on machine learning techniques, such as L1-regularized regression to identify and adjust for potential confounders efficiently within this high-dimensional space. This approach enables greater scalability, reduces reliance on manual variable selection, and supports reproducibility across studies.

LSPS has become the standard approach for confounding adjustment using propensity scores within distributed data networks like Observational Health Data Sciences and Informatics (OHDSI). OHDSI is a multi-stakeholder, interdisciplinary collaborative dedicated to bringing out the full value of observational health data through standardized data using a common data model (OMOP-CDM) and large-scale analytics. Within this network, LSPS has shown strong performance in large datasets and has been validated using negative controls to assess residual confounding. Importantly, LSPS also offers the benefit of adjusting for indirectly measured confounders, enhancing its robustness in causal effect estimation.^12^ Prior research also suggests LSPS outperforms manual covariate selection, as experts often choose different covariates and can miss important confounders identified by LSPS.^13^

However, it is unclear how well LSPS methods perform when sample sizes are limited. This is increasingly relevant as more observational studies are performed in federated network settings, where each participating site may have limited local data. Due to privacy concerns, it is a complex process to share and combine data. These datasets are also often from different populations (eg, different countries) and as such pooling the data is potentially inadvisable. This means that to maximize the utility of these data, analyses should be performed using a federated network approach. This means that understanding the quality of LSPS methods in smaller datasets is essential.

LSPS methods are typically evaluated using balance diagnostics to assess whether propensity score adjustment has achieved covariate balance between treatment groups, approximating randomization on measured variables. Standard diagnostics such as the standardized difference of means (SMD) compare covariate balance between treatment and comparator cohorts after PS adjustment and flag large imbalances. However, when applied to large numbers of covariates in small samples, this diagnostic can be misleading, indicating imbalance because of random error alone. A recent development aims to correct this problem by adding statistical testing to the SMD diagnostic and will be used here.^14^ While balance is an important diagnostic, treatment effect bias is a more direct and informative measure of whether the propensity score method is producing accurate effect estimates. Measuring bias reliably requires sufficient statistical power, which, often, a single small dataset cannot provide. This motived our study design of combining evidence across many small sites, enabling bias assessment with adequate power.

In practice, small datasets are often set aside because they appear insufficient on their own, even though they may still contain valid and valuable information. This is also a common issue for signal evaluation of newly marketed drugs, where there may be only small datasets available. If we have better methods, or if current methods prove to be working on small databases, we may be able to identify causal relationships earlier. Therefore, we aim to assess the reliability of LSPS in small-sample settings typical of distributed data networks. For this purpose, we emulate a distributed data network analysis by partitioning two large real-world databases, one electronic health record (EHR) and one claims database, into smaller and smaller subsets. We evaluate how well locally-fitted LSPS models perform in terms of covariate balance and bias measured using real negative and synthetic positive controls. We compare this to using a global LSPS model trained on the full data, and when no PS model is used at all in unadjusted analyses.

## Methods

To estimate the causal effect of the exposure choice on the outcome we use the new-user comparative cohort method, a standard approach within the Observational Health Data Sciences and Informatics (OHDSI) framework. In this approach, we compare patients who initiate one treatment (the target) to those who initiate another treatment (the comparator) using observational data.

### Data sources

To evaluate the performance of LSPS across different data environments, we selected one administrative claims database and one electronic health record (EHR) database. Specifically, we used the Merative MarketScan® Commercial Claims and Encounters (CCAE) database and the Optum® De-identified Electronic Health Record Dataset (Optum® EHR) dataset. Both databases were reviewed by the New England Institutional Review Board and were determined to be exempt from broad Institutional Review Board approval.

### Cohort definitions

Exposure cohorts were defined using a new-user design. Patients were eligible for inclusion upon their first recorded exposure to a study drug (index date), requiring at least 365 days of continuous prior observation before the index date and no concurrent membership in another treatment cohort. Each exposure cohort additionally required evidence of the relevant indication in the 365 days prior to index, specifically a diagnosis of hypertension (HTN), type 2 diabetes mellitus (T2DM), or major depressive disorder (MDD) depending on the drug class. Follow-up began at the index date and ended at treatment discontinuation, defined as a gap of more than 30 days in drug supply.

Outcome cohorts were derived from negative control outcomes; exposure-outcome pairs where no causal effect is expected,^15^ taken from the LEGEND studies.^16^

We conduct all analyses using the OHDSI CohortMethod package^17^ on data mapped to the Observational Medical Outcomes Partnership common data model (OMOP-CDM).^18^ We use a framework of empirically calculated diagnostics to assess power, potential measured confounding and residual confounding when performing this comparative cohort method using observational data.^19^

### Target-comparator selection

To ensure that the selected target-comparator (t-c) pairs exhibit confounding bias prior to adjustment, we first screen a large set of candidate comparisons. Using the CCAE database, we evaluate t-c pairs from our defined cohorts (HTN, T2DM, MDD), based on bias estimated in an unadjusted analysis from a large set of negative control outcomes. Where unadjusted analysis means, no method was applied for reducing bias. For each negative control, a Cox proportional hazard model was fitted with a single t-c indicator regressor. From this, we calculated, for the candidate comparisons, the level of bias, expressed as the lower bound of the expected absolute systematic error (EASElb). EASElb is defined as the lower bound of the 95% confidence interval on EASE.

The EASE estimates the expected magnitude of bias by fitting a Gaussian distribution to the estimated negative control hazard ratios,^15^ and then taking the expected absolute value of that distribution:

EASE = ∫|x|N(x; μ, σ)dx,

where N(x; μ, σ) is the Gaussian density function of *x* with mean μ and standard deviation σ, with μ and σ estimated using the negative controls. A low (0.0-0.1) EASElb indicates that there is little bias and a EASElb > 0.25 indicates that there is substantial bias.

To examine the impact of confounding adjustment across different scenarios, we selected six t-c, varying in levels of bias:

1. Losartan vs hydrochlorothiazide (EASElb = 0.26; high bias), with 76 negative controls (LEGEND-HTN^20^).
2. Quinapril vs propranolol (EASElb = 0.65; high bias), with 76 negative controls (LEGEND-HTN).
3. Glimepriride vs saxagliptin (EASElb = 0.38; high bias), with 94 negative controls (LEGEND-T2DM^21^).
4. Sitagliptin vs dapagliflozin (EASElb = 0.06; low bias), with 94 negative controls (LEGEND-T2DM).
5. Nortriptyline vs fluoxetine (EASElb = 0.51; high bias), with 52 negative controls (LEGEND-MDD).
6. Amitriptyline vs venlafaxine (EASElb = 0.31; high bias), with 52 negative controls (LEGEND-MDD).

### Outcomes

All negative outcomes are taken from the LEGEND studies as described above. Negative controls are specified as selected outcomes that are not believed to be caused or prevented by either treatment. When comparing the two treatments, we therefore assume that the hazard ratio of this negative control is equal to 1.

We additionally include synthetic positive controls, derived from the negative controls, to enable measuring type 2 error. Because unlike negative controls, we seldom know the true effect size of real positive controls, we employ synthetic positive controls, constructed by adding simulated outcomes to our real negative outcomes,^22^ achieving true effect sizes of 1.5, 2 and 4.

### Simulating smaller sites

For every t-c pair we then simulate a distributed data network analysis and assess LSPS performance in a small-sample, distributed setting, by randomly dividing the study population into smaller, equally sized, non-overlapping subsets (Figure 1). Within this framework, we compared three approaches: a *local* approach, in which a separate LSPS model was trained within each subset; a *global* approach, where a single PS model was trained on the full dataset and applied uniformly across all subsets; and an *unadjusted* approach, in which no PS adjustment was applied when fitting the outcome models. In the local and global propensity models, the PS adjustments applied are 1-1 matching and stratification (using 10 strata). Strata or matched sets with zero target or comparator subjects, or with no outcome events in either treatment group, do not contribute to the outcome model likelihood and are therefore excluded when fitting the model.

**Figure 1.**
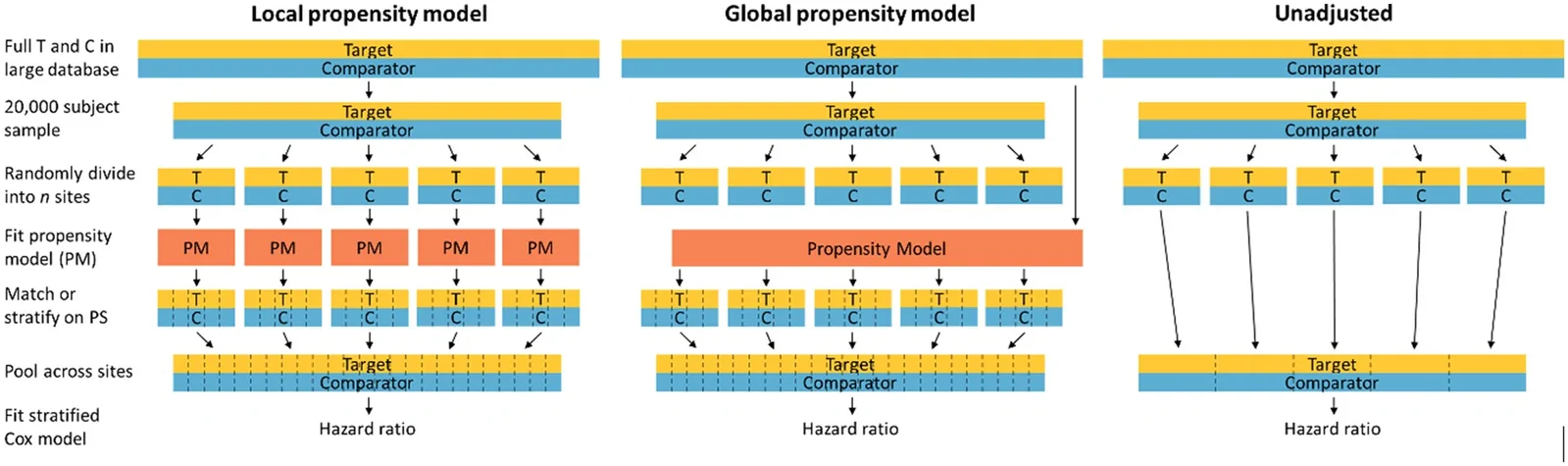
Simulating small data sites. We extract a target (T) and comparator (C) cohort from a large database and take a 20 000-person random sample. We then randomly divide these into k equally-sized sites. We evaluate propensity score adjustment using propensity models (PM) fitted at each simulated site (local) or using a single LSPS model fitted on the original full data (global) and compare this to no LSPS adjustment (unadjusted). Data is pooled across simulated sites before fitting a stratified Cox model.

We started with the full set of eligible patients. These were individuals who began one of the treatments of interest, had at least 365 days of prior observation and were not in both treatment groups. From this group, we randomly sampled 20 000 people. We then randomly divide (without replacement) the 20 000 patients once into *k* =

- 5 sites of 4000 persons
- 10 sites of 2000 persons
- 20 sites of 1000 persons
- 40 sites of 500 persons
- 80 sites of 250 persons

This approach allows us to assess how splitting data into smaller subsets impacts the ability of local propensity score models to account for confounding, while still maintaining overall power to evaluate bias.

### Propensity score adjustments

PS are computed using L1-regularized logistic regression,^23^ where the regularization hyperparameter is selected by optimizing out-of-sample likelihood in a 10-fold cross-validation. We compare treatment effect estimates under PS computed within each simulated site (*local*) and using the full population (*global*).

For the local PS analysis, the PS model was fitted separately within each simulated site using only that site’s data. PS matching (1:1), using a caliper of 0.2 on the logit scale,^5^ or stratification into 10 equally sized strata was then performed within each site. The resulting matching and stratification information was subsequently pooled across sites, and a Cox proportional hazards model was fitted on the pooled data to estimate the hazard ratio.

For the global PS analysis, the PS model was fitted on the full pooled population. The global PS scores were then applied to each site individually, after which matching and stratification were performed within site. The remaining steps followed the same procedure as the local analysis. The global analysis serves as a benchmark informing on any reduction in performance that is due to the fact that, as databases become smaller, it becomes less likely an appropriate match for a patient exists within the same database.

For the unadjusted analysis, no propensity score adjustment was applied, allowing evaluation of the degree of confounding when no adjustment is applied.

### Evidence synthesis

In many real-world settings, each site may only share summary-level results to be meta-analyzed. Methods for dealing with small counts when performing meta-analysis exist,^24^ but here we are primarily interested in assessing the small sample size performance of LSPS and therefore do not concern ourselves with how meta-analysis might also impact the quality of overall treatment effect estimate. We therefore forgo meta-analysis and pool person-level data from each site in fitting outcome models. These models do condition on the site. For PS stratification no PS site-strata are merged, resulting in a total of *k ×* 10 strata in the model when there are *k* sites.

### Metrics

We evaluated performance of treatment-effect estimates across four metrics, calculated using the HRs, each reflecting a distinct aspect of confounding control:

- **EASE** as explained before
- **Geometric mean of the precision** (1/(standard error)^2) of the log HR estimate after empirical calibration,^25^ where empirical calibration adjusts standard errors using negative and positive controls to correct for residual systematic error. Geometric mean of the precision after empirical calibration then reflects statistical precision; higher values indicate narrower confidence intervals for effect estimates.
- **Type I error** for negative controls, how often the null was rejected (at α = 0.05).
- **Type II error** for positive controls, how often the null was not rejected (at α = 0.05).
- **Maximum standardized difference of mean (SDM)** is computed by dividing the difference between the mean in T and C by the standard deviation for each covariate and taking the maximum of the absolute value. This measures the largest imbalance in baseline covariates between treated and comparator groups. This is an overall estimate calculated after all sites are pooled back together.
- **SMD significance test** as proposed by Hripcsak et al.^14^ to assess balance under small-sample conditions. A statistical test assessing whether standardized differences in covariates significantly exceed a prespecified threshold (we use 0.1), adjusting for sample size. It addresses the limitations of traditional SDM in small-sample settings by distinguishing meaningful imbalance from chance variation. This is an overall estimate calculated after all sites are pooled back together.

### Code availability

The code implementing the analyses in our study is publicly available at https://github.com/ohdsi-studies/SmallSampleEstimationEvaluation and can be directly applied to any dataset mapped to the OMOP CDM.

## Results

### Illustrating the computation of performance metrics

LSPS was used to fit propensity models and compute PS for each comparison in the overall population, as well as within each sampled subset. For example, Figure 2 shows the propensity score distributions for a single comparison (losartan versus hydrochlorothiazide), in one database (Optum® EHR), in a single subset at each sample size. The top left panel shows the PS distribution in one of the 80 250-person samples, while the bottom right panel shows the PS distribution of the large sample of 20 000 patients using the locally fitted LSPS models. In total, 6 clinical comparisons × 2 databases × (1 + 5 + 10 + 20 + 40 + 80 sampled subsets) = 1872 propensity models were fitted.

**Figure 2.**
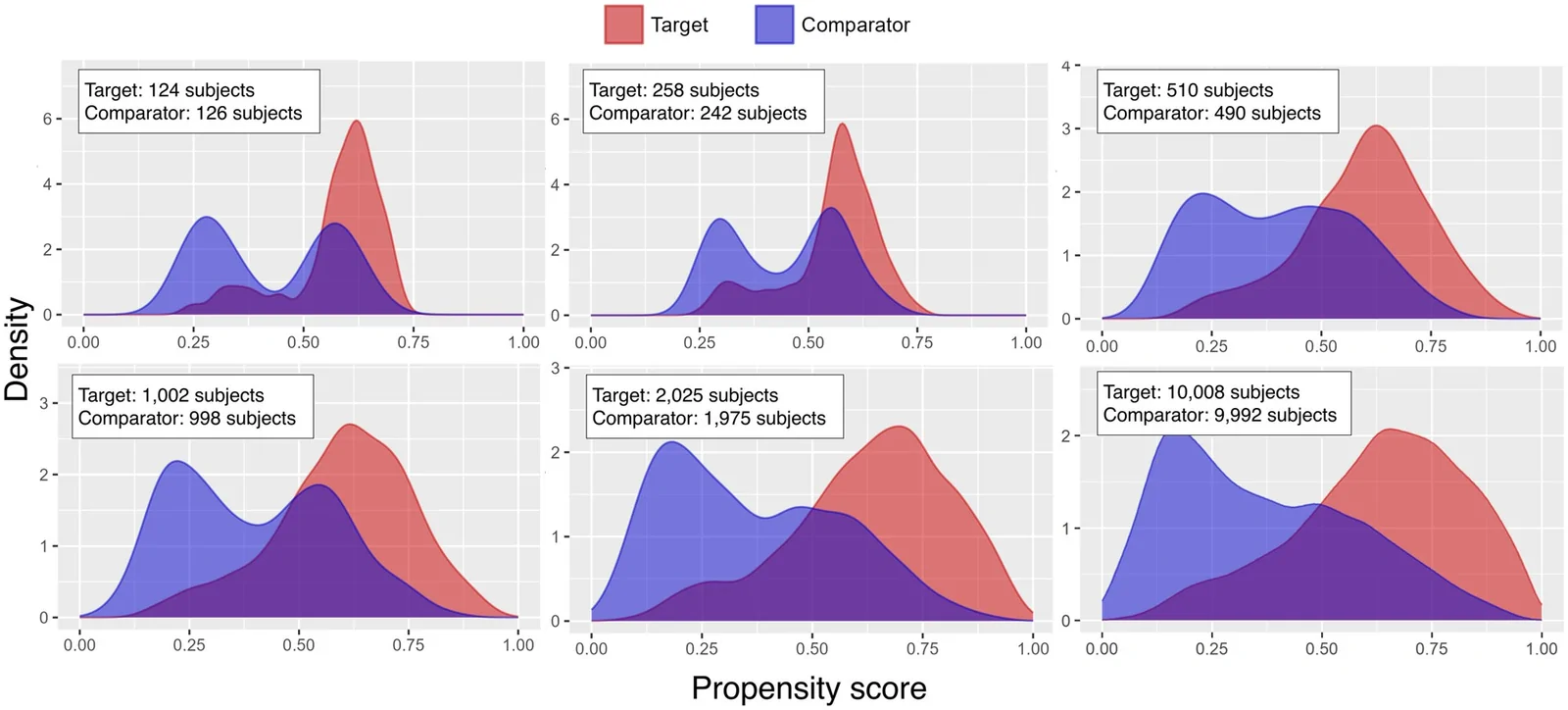
Propensity score distributions for t-c pair losartan versus hydrochlorothiazide, in a single subset using sample sizes 250, 500, 1000, 2000, 4000, and 20 000 using the locally fitted LSPS models.

### Computation of balance metrics

For computation of balance metrics, covariate balance between the treatment and comparator groups before and after matching are used. Supplementary Figure 1 shows an example of the covariate balance for losartan vs hydrochlorothiazide in the Optum® EHR database (for one subset at each sample size, using locally fitted LSPS models). We observe that the locally fitted LSPS-models on the bigger samples are more effective in improving covariate balances than LSPS models fitted on the smaller sites.

If we consider a standard rule-of-thumb that the maximum absolute SDM must be no greater than 0.1 to establish balance between the two populations, we observe (Figure S2) that we always fail this diagnostic when sample size per site is ≤ 4000 for both databases.

Following Hripcsak et al.,^14^ who showed that SMDs can be misleading in small samples, we apply their proposed SMD significance test. This method assesses whether observed SMDs significantly exceed a threshold (which we set at 0.1), accounting for sample size, and provides an overall count of significantly unbalanced covariates. As recommended, we apply a meta-analysis to the balance of each covariate.

Figure 3 shows that when applying the significance test, for most t-c pairs the number of significant unbalanced covariates is lower, and not all diagnostics will fail because low covariate balance. When increasing power by combining the smaller sites into a meta-analysis, as proposed by Hripcsak et al.^14^ for small sites, we even see that the number of significant unbalanced covariates is again lower. For some t-c pairs there are still a number of significant unbalanced covariates, that do increase with decreasing sample size.

**Figure 3.**
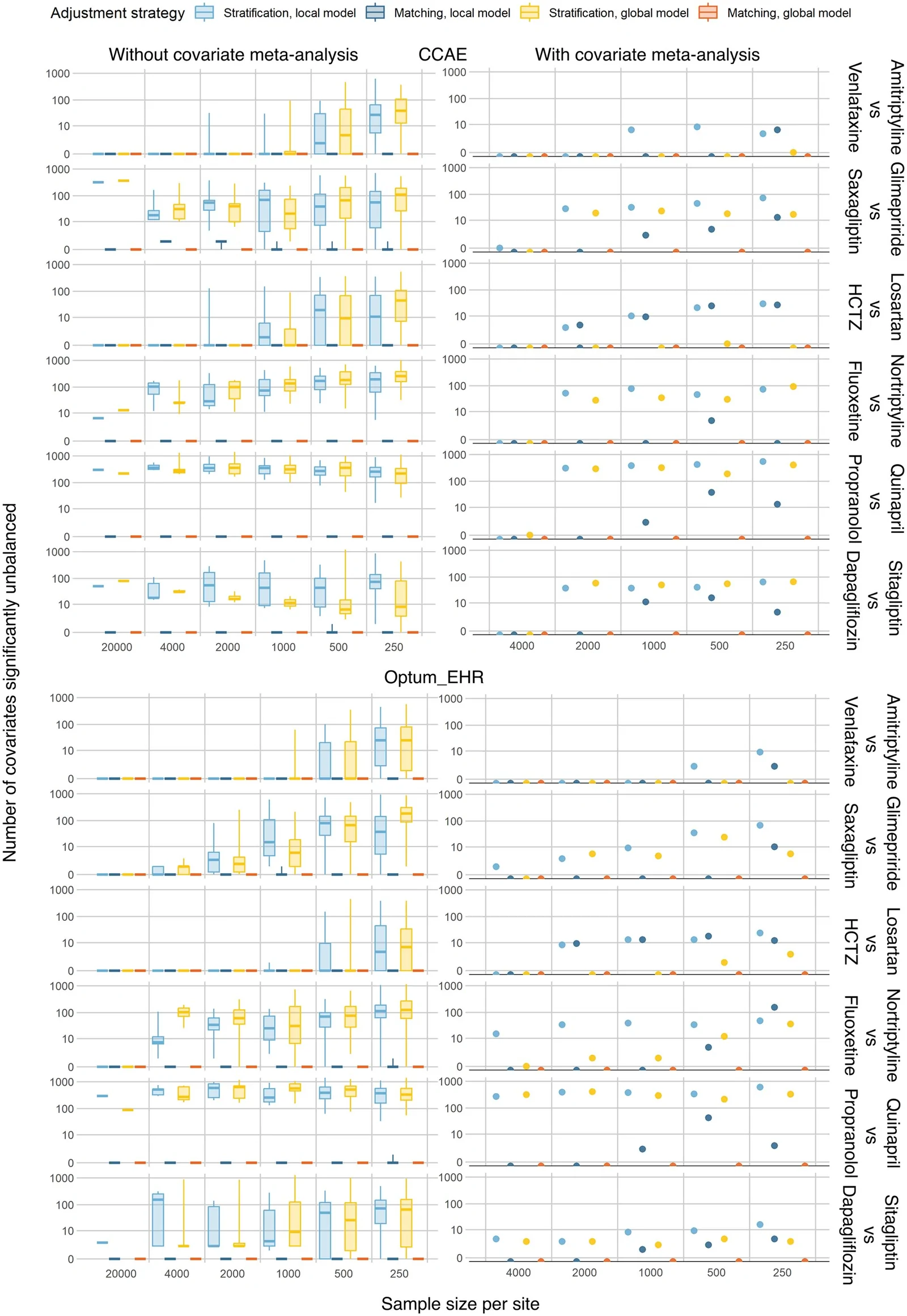
Significance test for unbalanced covariates with and without meta-analysis by sample size, based on the SMD significance test.

### Residual systematic error (EASE)

For each target-comparator-outcome, in each of the 2 databases and at each sample size, PS-matched, PS-stratified, and unadjusted datasets were pooled across sampled subsets. For example, Supplementary Figure 3 shows the estimates for a single comparison (losartan versus hydrochlorothiazide), in one database (Optum® EHR), by true effect size and sample size.

When extending the analysis to all target-comparator pairs and adjustment strategies, the EASE scores are summarized in Figure 4. In almost all cases, the application of adjustment methods reduced bias in terms of EASE compared to the unadjusted analyses. The one exception is for glimepriride vs saxagliptin in CCAE, where the EASE is already low without adjustment. While adjustment methods seemed to reduce bias in all pairs, still some pairs were left with substantial bias after adjustment, such as nortriptyline vs fluoxetine in the CCAE database. This effect is increasing with decreasing sample size. In certain cases, the global model appeared to reduce bias more effectively, though this pattern was neither strong nor consistent across all comparisons. In most scenarios, PS matching shows lower EASE scores than PS stratification. In fact, PS matching only rarely results in EASE > 0.2.

**Figure 4.**
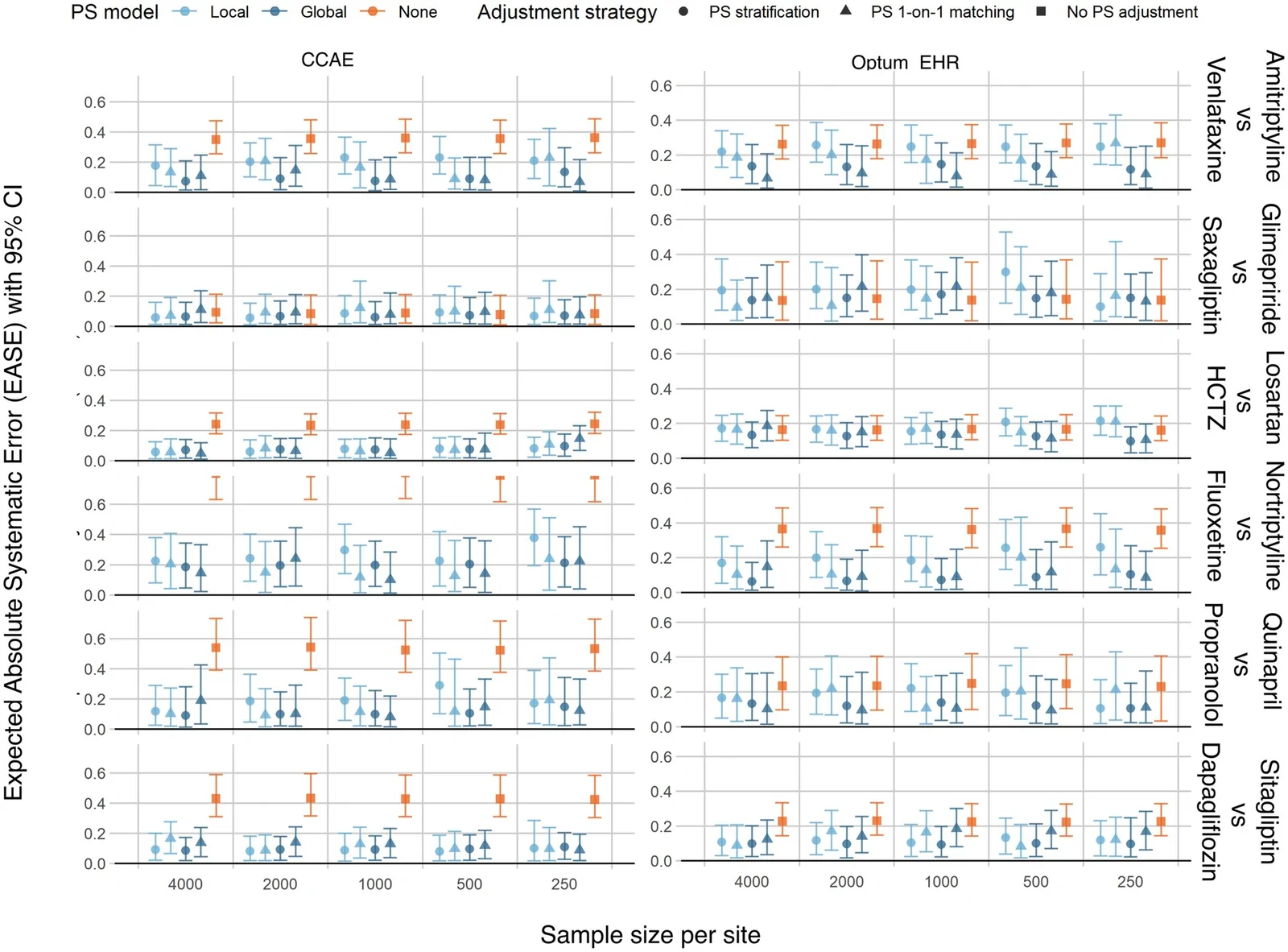
Expected Absolute Systematic Error (EASE) with 95% credible intervals per sample size.

### Precision after calibration

To disentangle the tradeoff between bias and precision, we examine the precision of the calibrated CI (Figure 5), alongside Type 1 and Type 2 error rates (Supplementary Figure 4). Precision and power are meaningful if the Type 1 error is near nominal.

**Figure 5.**
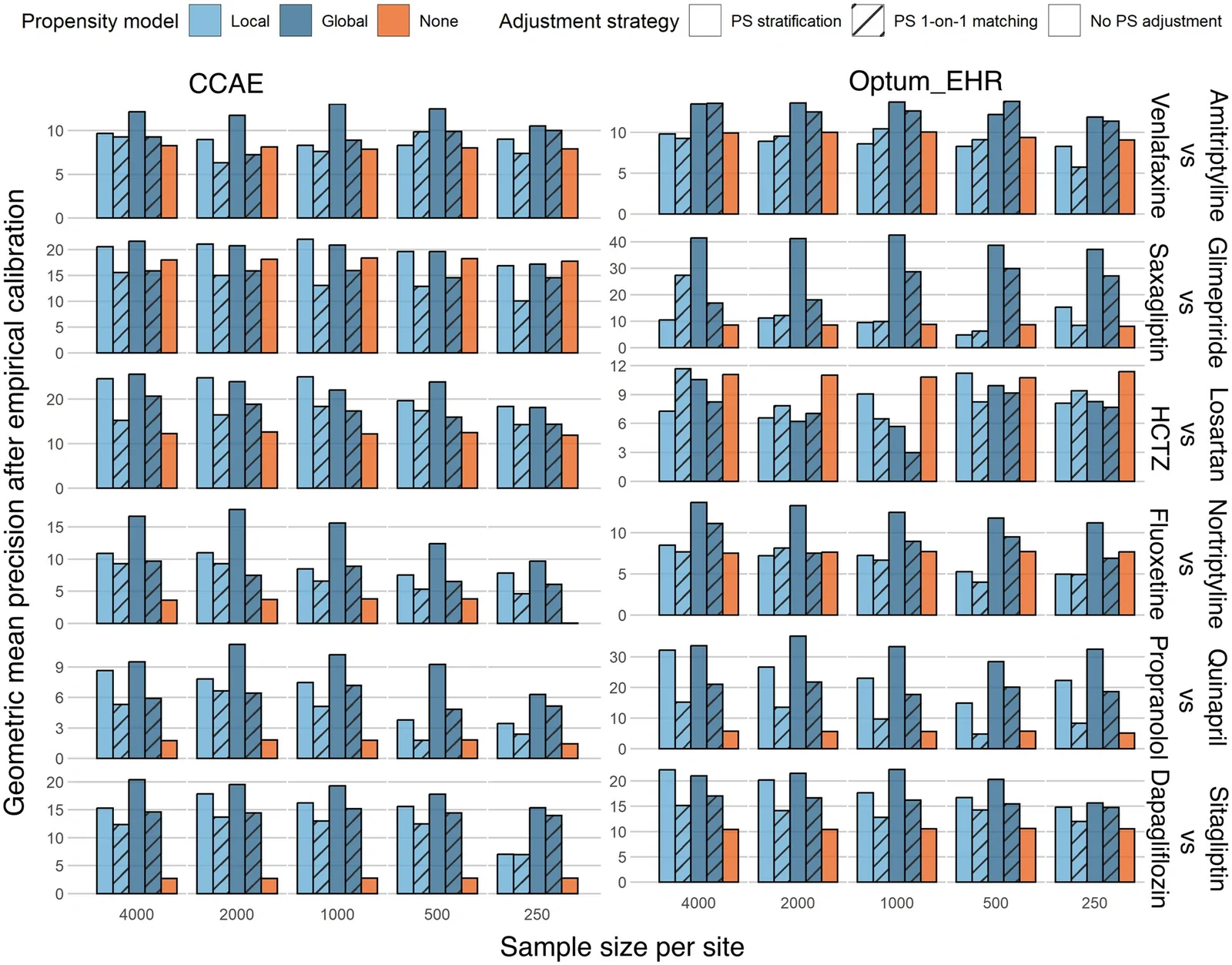
Geometric mean precision after empirical calibration based on both negative and positive control estimates.

Figure 5 shows that PS adjustment generally improves precision after empirical calibration across all target-comparator pairs, with the same two exceptions observed earlier: glimepiride vs saxagliptin in CCAE, and losartan vs HCTZ in Optum® EHR. The largest precision gains are seen with stratification, especially when using the global LSPS model. In some t-c pairs these gains decline as sample size decreases, particularly for the nortriptyline vs fluoxetine and quinapril vs propranolol comparisons. Type 1 error was generally well-controlled near the nominal 0.05 level across most adjustment strategies, though some variability was observed across t-c pairs and databases (quinapril vs propranolol in both databases). Losartan vs HCTZ is only t-c pair with both low type I and type II error for all adjustment strategies in both databases.

Power (1 − Type 2 error) was generally low across all comparisons and strategies, with Type 2 error frequently exceeding 0.25-0.50, even in the big sample sizes.

## Discussion

Our findings suggest that LSPS reduces confounding compared to no adjustment, even when sample sizes are very small, unless the unadjusted analyses are already unconfounded, in which case LSPS shows equal performance. In most cases, the local PS model shows similar performance to the global model, suggesting little degradation in the quality of the PS models, despite the need to fit quite high-dimensional models with less and less data. However, for some comparisons, in some databases, the confounding adjustment does become less effective as sample size decreases, although it is hard to pinpoint under which circumstances. PS matching appears to be superior to PS stratification in reducing bias as measured by EASE, and this finding is confirmed by the balance metric using significance testing. However, this reduced bias relative to stratification appears to come at the cost of precision, and if one were to use empirical calibration, stratification may be preferred as it produces the highest precision after calibration, with low type I error.

The standard way of estimating covariate balance by SDM always fails in our analyses when sample sizes are small. The new balance metric, where significantly unbalanced covariates are estimated, does not show this effect. This suggests that this metric is more robust to fairly evaluate covariate balance in smaller sites.

Even though LSPS seemed to reduce bias significantly in most t-c pairs, some pairs continued to have residual bias in the smaller sites. For these t-c pairs, alternative approaches may help address these challenges for sites with high residual bias, researchers can try to incorporate prior knowledge of confounders, apply dimensionality reduction, or use cardinality matching to see if they improve residual bias.^26^

In summary, these results suggest LSPS methods remain effective tools for confounding adjustment in many small-sample settings. Standard covariate balance diagnostics based on the standardized difference of means consistently fail in small samples and may therefore incorrectly suggest that these data are unusable. In contrast, a recently proposed balance metric that accounts for chance imbalance, indicate that many small sites still achieve adequate balance and can contribute meaningful information. Relying solely on SDM-based diagnostics risks discarding valuable data in situations where LSPS methods are capable of substantially reducing systematic bias.

At the same time, our findings also emphasize the need for careful application and additional methodological innovation in small-sample settings. Future research should focus on refining diagnostic tools and exploring alternative or hybrid modeling strategies to enhance causal inference robustness across varying data conditions. In addition, future work should evaluate large-scale propensity scores across truly independent databases and real federated networks to determine whether the findings observed here generalize to heterogeneous healthcare settings.

## Limitations

A limitation of our study is that we used two large national databases partitioned into smaller subsets to emulate a distributed data network. Because these simulated sites were created by randomly partitioning a single database, they do not reflect the population, coding, prescribing, and geographic differences that exist across real-world sites. While this design allows us to isolate the effect of sample size, it does not capture the full heterogeneity of real-world networks, such as differences in patient populations, treatment patterns, and coding practices across sites. Future studies could address this by combining multiple distinct databases or using state-level partitions to better reflect site-specific variation. Despite this limitation, our approach provides a controlled framework to evaluate LSPS performance and yields valuable insights into the impact of sample size on confounding adjustment.

## Supplementary Material

ocag133_Supplementary_Data

## Data Availability

The data underlying this article cannot be shared publicly due to the privacy of individuals whose data was used in the study. Aggregated data to support the findings of this study can be made available upon a reasonable request from the corresponding author.
